# NLRP6 Plays an Important Role in Early Hepatic Immunopathology Caused by *Schistosoma mansoni* Infection

**DOI:** 10.3389/fimmu.2020.00795

**Published:** 2020-05-05

**Authors:** Rodrigo C. O. Sanches, Cláudia Souza, Fabio Vitarelli Marinho, Fábio Silva Mambelli, Suellen B. Morais, Erika S. Guimarães, Sergio Costa Oliveira

**Affiliations:** ^1^Departamento de Bioquímica e Imunologia, Instituto de Ciências Biológicas, Universidade Federal de Minas Gerais, Belo Horizonte, Brazil; ^2^Departamento de Genética, Ecologia e Evolução, Instituto de Ciências Biológicas, Universidade Federal de Minas Gerais, Belo Horizonte, Brazil; ^3^Instituto Nacional de Ciência e Tecnologia em Doenças Tropicais (INCT-DT), CNPq MCT, Salvador, Brazil

**Keywords:** *Schistosoma mansoni*, immunopathology, inflammasome, NLRP6, fibrosis

## Abstract

Schistosomiasis is a debilitating parasitic disease that affects more than 200 million people worldwide and causes approximately 280,000 deaths per year. Inside the definitive host, eggs released by *Schistosoma mansoni* lodge in the intestine and especially in the liver where they induce a granulomatous inflammatory process, which can lead to fibrosis. The molecular mechanisms initiating or promoting hepatic granuloma formation remain poorly understood. Inflammasome activation has been described as an important pathway to induce pathology mediated by NLRP3 receptor. Recently, other components of the inflammasome pathway, such as NLRP6, have been related to liver diseases and fibrotic processes. Nevertheless, the contribution of these components in schistosomiasis-associated pathology is still unknown. In the present study, using dendritic cells, we demonstrated that NLRP6 sensor is important for IL-1β production and caspase-1 activation in response to soluble egg antigens (SEA). Furthermore, the lack of NLRP6 has been shown to significantly reduce periovular inflammation, collagen deposition in hepatic granulomas and mRNA levels of α-SMA and IL-13. Livers of *Nlrp6*^–/–^ mice showed reduced levels of CXCL1/KC, CCL2, CCL3, IL-5, and IL-10 as well as Myeloperoxidase (MPO) and Eosinophilic Peroxidase (EPO) enzymatic activity. Consistently, the frequency of macrophage and neutrophil populations were lower in the liver of NLRP6 knockout mice, after 6 weeks of infection. Finally, it was further demonstrated that the onset of hepatic granuloma and collagen deposition were also compromised in *Caspase-1^–/–^*, *IL-1R^–/–^* and *Gsdmd*^–/–^ mice. Our findings suggest that the NLRP6 inflammasome is an important component for schistosomiasis-associated pathology.

## Introduction

Schistosomiasis is a debilitating parasitic disease which affects 78 countries worldwide. This disease leads to approximately 200,000 deaths annually and severely compromises the life quality from those affected. Among causative species, *Schistosoma haematobium*, *Schistosoma japonicum*, and *Schistosoma mansoni* stand out as those of major importance to human health (1–3). Infection occurs through the direct contact of the host with the parasite’s larval form. After parasite penetration and sexual development, egg laying begins. The release of eggs in the feces and its hatching in the environment closes the parasite’s life cycle (4). However, a significant amount of these eggs is trapped in some of the host’s organs, such as liver and intestine, where they induce a granulomatous inflammatory reaction (5, 6). Hepatic granulomatous inflammation arises from the egg-secreted antigens, which perform hepatotoxic and immunological activities capable of recruiting immune cells to the organ and forming periovular granuloma. The composition of the granuloma includes macrophages, eosinophils, neutrophils, T and B lymphocytes and especially fibroblasts, responsible for the fibrotic characteristic of the structure (7, 8).

Although the process of hepatic granuloma formation is extensively studied, all cellular events and key participants have not been fully established yet. The role of intracellular immune receptors in granuloma formation, for instance, was first described in a seminal study conducted by Ritter and colleagues (9). However, a better understanding of cytosolic sensors during *S. mansoni* infection is required. These intracellular receptors are those responsible for activating the inflammasome pathway. This pathway induces the formation of an intracellular protein complex typically consisting of Nucleotide-binding oligomerization domain (NOD), leucine-rich repeat (LRR)- containing protein (NLR) family members, an adapter molecule known as ASC, and the cysteine protease caspase-1 as an effector molecule. Activation of this pathway leads to cleavage of immature forms of IL-1β and IL-18 into their mature forms. It might also induce cell death by pyroptosis. The inflammasome activation takes place in both immune and non-immune cells and is essentially triggered by pathogen-associated molecular patterns (PAMPs) and Danger-associated molecular patterns (DAMP) (10, 11).

It is known that the inflammasome pathway plays an important role during chronic liver diseases (12). Besides fighting pathogens such as bacteria (13) and viruses (14), inflammasome also participates in aggravating sterile liver inflammations such as Alcoholic Liver Disease (ALD) (15) and Non-alcoholic Steatohepatitis (NASH) (16). NLRP3 is the most widely studied receptor in this context since it is activated by several types of insults (17). On the other hand, the participation of other NLR family receptors in hepatic pathological processes, such as NLRP6, is still elusive.

The inflammasome pathway plays an essential role in schistosomiasis-associated liver pathology. It has been demonstrated that NLRP3 is critical for granuloma formation and hepatic stellate cells (HSCs) activation (18) in *S. japonicum* infections. Regarding *S. mansoni* infection this same sensor has been shown to be involved in the adaptive immune response and also granuloma formation (9). Recent studies have reported that NLRP3 and NLRP6 expression are simultaneously modulated in some processes, including those occurring in the liver (19, 20). Additionally, the role of NLRP6 in fibrotic diseases has already been described (21, 22). Thus, we decided to investigate whether the NLRP6 sensor plays a role in the course of *S. mansoni* infection and liver pathology. In this study, we demonstrate that lack of NLRP6 modulates the formation of hepatic granuloma, influencing local chemokine and cytokine production as well as macrophage and neutrophil recruitment into the liver. Also, this receptor is important for promoting collagen deposition.

## Materials and Methods

### Ethics Statement

This study was carried out in accordance with Brazilian laws #6638 and #9605 in Animal Experiments. The protocol was approved by the Committee on Ethics of Animal Experiments of the Universidade Federal de Minas Gerais (UFMG) (Permit Number: #367/2017).

### Mice and Parasite

Wild-type C57BL/6 mice were purchased from the Universidade Federal de Minas Gerais (UFMG). *Nlrp3*^–/–^, *Nlrp6*^–/–^, *Casp-1^–/–^*, *IL-1R^–/–^*, and *Gsdmd*^–/–^ were described previously (23–26). The animals were maintained at UFMG and used at 6–10 week of age. *Schistosoma mansoni* (LE strain) cercariae at Fundação Oswaldo Cruz – Centro de Pesquisas René Rachou (CPqRR-Brazil) were routinely obtained from infected *Biomphalaria glabrata* snails exposed to light, inducing the shedding of parasites.

### Eggs, SEA, and SWAP

Eggs were obtained from 50-day-infected Swiss mice livers. Briefly, the liver was blender processed in cold saline (2% NaCl) for 2 min. Next, the material was decanted into a glass goblet for 35 min at low temperature. Part of the decanting supernatant was discarded and the remaining solution was washed with cold saline. Decantation-washing was repeated until reaching a translucent solution. Eggs were recovered by filtration. For the preparation of Soluble Egg Antigens (SEA), eggs were disrupted for 40 min at low temperature in PBS and then the homogenate was centrifuged at 100,000 × *g* for 1 h at 4°C. The resulting supernatant was frozen at −80°C. Soluble adult worm antigen (SWAP) was obtained by mechanical maceration of worms in cold PBS. After centrifugation (13,000 × *g* – 7 min), the supernatant was collected and stored at −80°C. The protein concentration of SEA and SWAP was determined using BCA^TM^ protein assay kit (Thermo Fisher Scientific, Waltham, MA, United States).

### BMDC Generation and Activation

To obtain bone marrow-derived dendritic cells (BMDCs), bone marrow cells were cultured in RPMI with 10% FBS, 100 U/mL penicillin, 100 μg/mL streptomycin and 20 ng/mL murine recombinant GM-CSF (Peprotech, Riberão Preto, Brazil). Petri dishes containing 1 × 10^7^ cells were incubated at 37°C in 5% CO_2_. At day 3 of incubation, 5 mL of fresh complete medium with GM-CSF was added, and 5 mL of medium was replaced with fresh supplemented medium containing GM-CSF on days 5 and 7. At day 10, non-adherent cells were harvested and seeded in 24-well plates (5 × 10^5^ cells/well). Stimulation of BMDCs was performed by priming cells with 1 μg/ml of Pam_3_Cys (Sigma-Aldrich, St. Louis, MO, United States) for 5 h and then stimulating with 50 μg/mL of SEA for 17 and 24 h. As positive control for inflammasome activation, cells were primed with 1 μg/ml of Pam_3_Cys (5 h) or 1 μg/ml of LPS (4 h) and stimulated with ATP (5 mM) (50 min) or Nigericin (20 μM) (50 min). Culture supernatants were collected and cells were lysed with M-PER Mammalian Protein Extraction Reagent (Thermo Fisher Scientific) supplemented with 1:100 protease inhibitor mixture (Sigma-Aldrich).

### Western Blotting

Cell lysates and supernatants from DCs culture were subjected to SDS-PAGE analysis and western blotting. The proteins were resolved on a 15% SDS-PAGE gel, and transferred to nitrocellulose membranes (Amersham Biosciences, Uppsala, Sweden). Membranes were blocked for 1 h in TBS (0.1% Tween-20; 5% non-fat dry milk) and incubated with primary antibodies at 4°C, overnight. Primary antibody used was mouse monoclonal against the p20 subunit of caspase-1 (Adipogen, San Diego, CA, United States). Monoclonal antibody against β-actin (Cell Signaling Technology, Danvers, MA, United States) was used as a loading control blot (1:1,000). The membranes were washed three times for 10 min in TBS with 0.1% Tween 20. Next, membranes were incubated for 1 h at room temperature with the suitable HRP-conjugated secondary antibody (1:1,000). Immunoreactive bands were visualized using Luminol chemiluminescent HRP substrate (Millipore).

### Splenocyte Culture

Spleen cells were obtained from macerated spleens of individual C57BL/6 and *Nlrp6*^–/–^ mice after 6 weeks of infection with *S. mansoni* cercariae (*n* = 5/group). Cells were washed with PBS and the erythrocytes were lysed with a hemolytic solution (155 mM NH4Cl, 10 mM KHCO3, pH 7.2). Cells were adjusted to 1 × 10^6^/well in complete RPMI medium (10% fetal bovine serum, 100 U/mL penicillin and 100 μg/mL streptomycin). Spleen cells were cultured in 96-well plates with medium and stimulated with SWAP (200 μg/mL), SEA (20 μg/mL), Eggs (50/well) or concanavalin A (ConA) (5 μg/mL). Culture supernatants were collected after 24 h for IL-5 and after 72 h for IFN-γ, IL-10, and IL-13 measurements by ELISA.

### Liver Processing

Right lobe of liver from 6-week-infected C57BL/6 and *Nlrp6*^–/–^ mice was collected and 1 mL of cytokine extraction solution (0.4 M NaCl, 0.05% Tween 20, 0.5% BSA, 0.1 mM PMSF, 0.1 mM benzethoniumchloride, 10 mM EDTA and 20 KI aprotinin) was added to each 100 mg of tissue. Ultra-Turrax homogenizer-dispenser was used to homogenize solutions containing the organs. Next, the samples were centrifuged at 10,000 × *g* for 10 min at 4°C. Non-parenchymal cells from left lobe were used for flow cytometry analysis. Tissue was removed without perfusion, cut into small pieces, incubated in RPMI medium containing 30 μg/ml of Liberase TM (Roche) and 20 U/mL of DNAse I (GE) for 40 min, and passed through a 70 μm pore-size cell strainer. After centrifugation, the cells were resuspended in PBS containing 2% fetal bovine serum (FBS) and 5 mM EDTA. Low-speed centrifugation (50 × *g* – 5 min) was used to remove parenchymal cells. Erythrocytes were lysed with a hemolytic solution (155 mM NH_4_Cl, 10 mM KHCO_3_, pH 7.2). The remaining non-parenchymal cells were resuspended in RPMI culture medium.

### Cytokine Measurements

Cytokine/Chemokine production was evaluated using the Duoset ELISA kit (R&D Diagnostic, Minneapolis, MN, United States) according to the manufacturer’s instructions.

### EPO and MPO Activity Assays

Eosinophilic Peroxidase and Myeloperoxidase assays were performed as described by Cançado et al. (27). Right lobe of the liver was homogenized, red blood cells subjected to hypotonic lysis and the remaining liver cells subjected to detergent lysis and freeze-thaw cycles. The enzymatic assay was performed using the suitable substrates and the result was measured on a microplate reader at the appropriate wavelength (492 nm for EPO and 450 nm for MPO). The result was expressed in absorbance units.

### Flow Cytometry Analysis

Spleen and non-parenchymal liver cells were stained for CD11b, CD11c, Ly6G, F4/80, CD3, and CD4. Briefly, cells were incubated for 20 min with anti-mouse CD16/32 (BD Biosciences) in FACS buffer (PBS, 1% FBS, 1 mM NaN3) and were stained for surface markers for 20 min using: APC-Cy7-conjugated anti-mouse CD11b (1:200, M1/70; BD Biosciences), FITC-conjugated anti-mouse CD11c (1:100, HL3; BD Biosciences), PE-conjugated anti-mouse Ly6G (1:200, 1A8; BD Biosciences), biotinylated anti-mouse F4/80 (1:200, BM8; BD Biosciences), PE-Cy7-conjugated anti-mouse CD3 (1:100, BD Biosciences) and APC-conjugated anti-mouse CD4 (1:200, BD Biosciences). The appropriate isotype controls were used. Next, cells were washed and incubated for 20 min at 4°C in the dark with PerCP-Cy5.5 conjugated streptavidin (1:200 BD Biosciences). Lastly, cells were washed and resuspended in PBS. Attune Flow Cytometer (Applied Biosystems, Waltham, MA, United States) was used for collecting approximately 100,000 events and data were analyzed using FlowJo software (Tree Star, Ashland, OR, United States).

In order to evaluate macrophage polarization, non-parenchymal liver cells were stained as described above using APC-Cy7-conjugated anti-mouse CD11b (1:200, M1/70; BD Biosciences), biotinylated anti-mouse F4/80 (1:200, BM8; BD Biosciences), BB700-conjugated anti-mouse CD197 (1:200, 4B12 BD Biosciences), FITC-conjugated anti-mouse CD80 (1:200, 16-10A1, BD Biosciences), PE-conjugated anti-mouse CD163 (1:200, TNKUPJ, eBioscience) and APC-conjugated anti-mouse CD206 (1:200, MR5D3, BD Biosciences).

### Quantitative Real-Time PCR

Liver middle lobe of 6-week-infected C57BL/6 and *Nlrp6*^–/–^ mice was used for RNA extraction. The tissue was homogenized in TRIzol (Invitrogen) and total RNA was isolated in accordance with the manufacturer’s instructions. Reverse transcription of total RNA was performed and quantitative real-time RT-PCR was conducted in a final volume of 20 μL containing SYBR Green PCR Master Mix (Applied Biosystems, Foster City, CA, United States), oligo-dT cDNA as the PCR template and 2.5 μM of primers. The PCR reaction was performed with QuantStudio3 real-time PCR instrument (Applied Biosystems). Primers were used to amplify a specific fragment (100–120 bp) corresponding to specific gene targets as follows: *18S* Forward (5′-CGTTCC ACCAACTAAGAACG-3′) and *18S* Reverse (5′-CTCAACACGG GAAACCTCAC-3′; α*-SMA* Forward (5′-GTCCCAGACATCAG GGAGTAA-3′) and α*-SMA* Reverse (5′-TCGGATACTTCA GCGTCAG-3′); *IL-13* Forward (5′-CCTGGCTCTTGCTTGCC-3′) and *IL-13* Reverse (5′-GGTCTTGTGTGATGTTGCTCA-3′); *IL-1*β Forward (5′-TGACCTGGGCTGTCCAGATG-3′) and *IL-1*β Reverse (5′-CTGTCCATTGAGGTGGAGAG-3′); *Casp-1* Forward (5′-GGAAGCAATTTATCAACTCAGTG-3′) and *Casp-1* Reverse (5′-GCCTTGTCCATAGCAGTAATG-3′).

### Mice Infection and Parasite Burden

Six-to-eight-week-old wild-type and knockout mice were anesthetized with 5% ketamine, 2% xylazine and 0.9% NaCl and then infected with 100 cercariae (LE strain) through exposure of percutaneous abdominal skin, for 1 h. After 6 weeks of infection, mice were euthanized and perfused from the portal veins, the recovered worms were counted and the mean difference between groups of mice was evaluated.

### Pathological Parameters

Number of eggs was obtained from liver median lobe. The tissue was weighed and digested in an aqueous solution of KOH (5%) for 16 h at 37°C. After, eggs were washed in saline and centrifuged twice at 270 × *g* for 10 min and counted using a light microscope. The number of calculated eggs was corrected by considering the mass of the tissue, resulting in number of eggs per gram of liver. The left lobe was fixed with 10% buffered formaldehyde in PBS. Histological sections were performed using microtome at 6 μm and stained on a slide with Hematoxylin-Eosin (HE) and Masson blue. For measurement of granuloma size and collagen deposition, a JVC TK-1270/RBG camera, attached to the microscope (10 × objective lens), was used to obtain the images. Analysis were carried out using ImageJ software (U.S. National Institutes of Health, Bethesda, MD, United States)^1^. Granuloma size was measured, in μm^2^, for all granulomas found in liver sections.

### Statistical Analysis

The statistical tests were performed using Student’s *t-test*, one-way and two-way ANOVA followed by Bonferroni adjustments for comparison between groups. *P*-values obtained were considered significant if they were <0.05. Statistical analysis was performed using GraphPad Prism 6 (La Jolla, CA, United States).

## Results

### IL-1β Production and Caspase-1 Activation Are Partially Dependent on NLRP6 in SEA/Eggs-Stimulated Dendritic Cells

**FIGURE 1:**
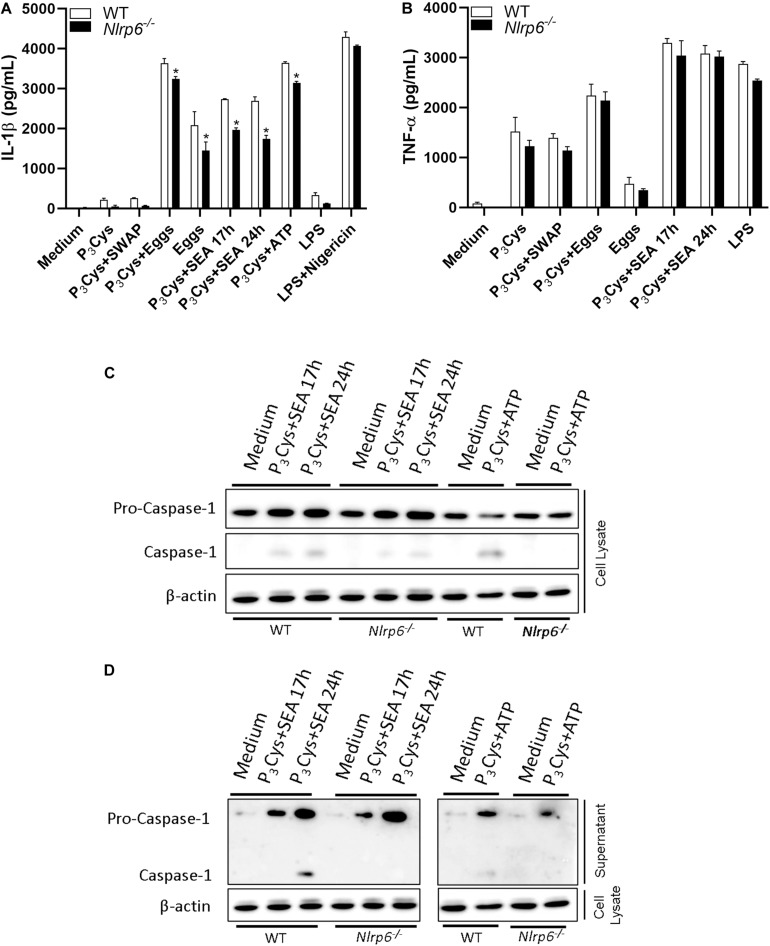
NLRP6 regulates IL-1β production and Casp-1 activation induced by SEA. WT or *Nlrp6*^–/–^ deficient BMDCs were primed with P_3_Cys (1 μg/ml – 5 h) and stimulated with SEA (50 μg/mL – 17 h, 24 h), Eggs (100 eggs/well – 24 h), SWAP (200 μg/mL – 24 h) or ATP (5 mM – 50 min). For nigericin control (20 μM), cells were primed with LPS (1 μg/ml – 4 h) and stimulate for 50 min. **(A)** IL-1β and **(B)** TNF-α were measured by ELISA. Casp-1 activation was analyzed by western blot in **(C)** cell lysate and **(D)** supernatant using antibody against p20 subunit. An asterisk denotes statistically significant differences between NLRP6 versus WT animals (*p* < 0.05).

During *S. mansoni* infection, hepatic dendritic cells (DCs) are the main cells responsible for promoting the shift from Th1 to Th2 immune profile, which is triggered by egg antigens (28). Ritter and colleagues (2010) reported that SEA induces inflammasome activation in DCs and described the involvement of NLRP3 (9). Thus, given the relevance of these cells, we decided to use bone marrow-derived dendritic cells (BMDCs) in order to investigate whether the NLRP6 sensor is involved in IL-1β secretion in response to different parasite antigens. Using Pam_3_Cys (P_3_Cys) as the first signal, we observed that eggs and their soluble antigens (SEA) induce high levels of IL-1β and this production was partially influenced by NLRP6 (Figure 1A). In contrast, IL-1β production induced by soluble adult worm antigens (SWAP) was much lower compared to SEA (Figure 1A). Additionally, TNF-α levels were not altered comparing both WT and NLRP6 knockout (KO) mice (Figure 1B). Since SEA was sufficient to induce IL-1β production, we used this stimulus to evaluate the role of NLRP6 in caspase-1 (casp-1) activation. Figures 1C,D demonstrate that SEA induces activation of casp-1 in WT DCs and this process was clearly inhibited in *Nlrp6*^–/^*^–^* DCs.

### NLRP6 Influences Granuloma Formation and Collagen Deposition in the Liver

**FIGURE 2:**
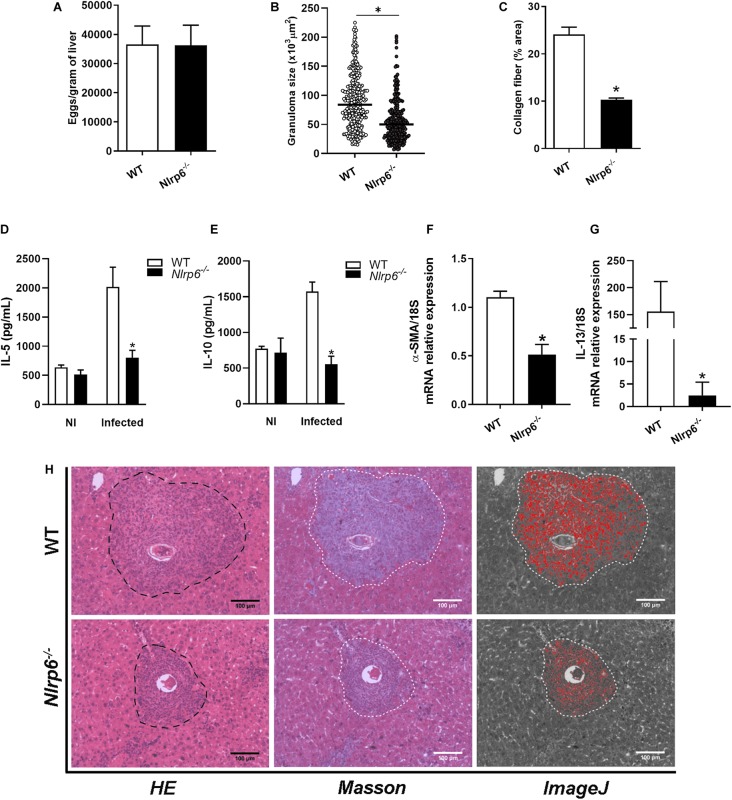
*Schistosoma*-induced liver pathology is influenced by NLRP6 sensor. Pathological and molecular parameters were evaluated, after 6 weeks of infection in WT and *Nlrp6*^–/–^ mice. **(A)** Number of eggs per gram of liver, **(B)** Granuloma size (μm^2^) and **(C)** Collagen deposition. **(D)** IL-5 and **(E)** IL-10 cytokine levels were detected. **(F)** α-SMA and **(G)** IL-13 transcripts were also measured. **(H)** Representative images of granulomas detected in hematoxylin-eosin, masson blue and the output from ImageJ software, respectively. An asterisk denotes statistically significant differences between NLRP6 versus WT animals (*p* < 0.05). The bar represents 100 μm. NI stands for Non-infected mice.

Since the liver is one of the main entrapment tissues for parasite’s eggs and once the participation of NLRP6 in the egg and SEA-induced immune response has been observed, we wondered if this sensor could play any role in liver pathology. First, we observed that in livers of 4- and 6-week infected animals the levels of *IL-1*β and *caspase-1* mRNA did not significantly change between *Nlrp6*^–/–^ and WT mice (Supplementary Figure S1). Initially, we observed that the number of eggs per gram of tissue was not altered between WT and knockout mice (Figure 2A). Consistently, the worm burden recovery was the same comparing both groups (Supplementary Figure S2). On the other hand, NLRP6 has been shown to influence the periovular inflammatory response, contributing significantly to granuloma formation (Figure 2B). In addition, collagen deposition within the granulomatous structure was reduced in *Nlrp6*^–/–^ mice when compared to WT (Figures 2B,C,H). We also evaluated levels of cytokines and fibrotic markers within the tissue, such as the cytokines IL-5, IL-10, IL-13, and the protein Alpha-smooth muscle actin (α-SMA). IL-5 and IL-10 cytokines levels, as well as, α-SMA and IL-13 mRNA measurements were reduced in *Nlrp6*^–/–^ group when compared to WT (Figures 2D–G). These data demonstrate that NLRP6 contributes to the pathology caused by *S. mansoni*.

### NLRP6 Influences IL-10 and IFN-γ Production by Spleen Cells Activated With Egg Antigens

**FIGURE 3:**
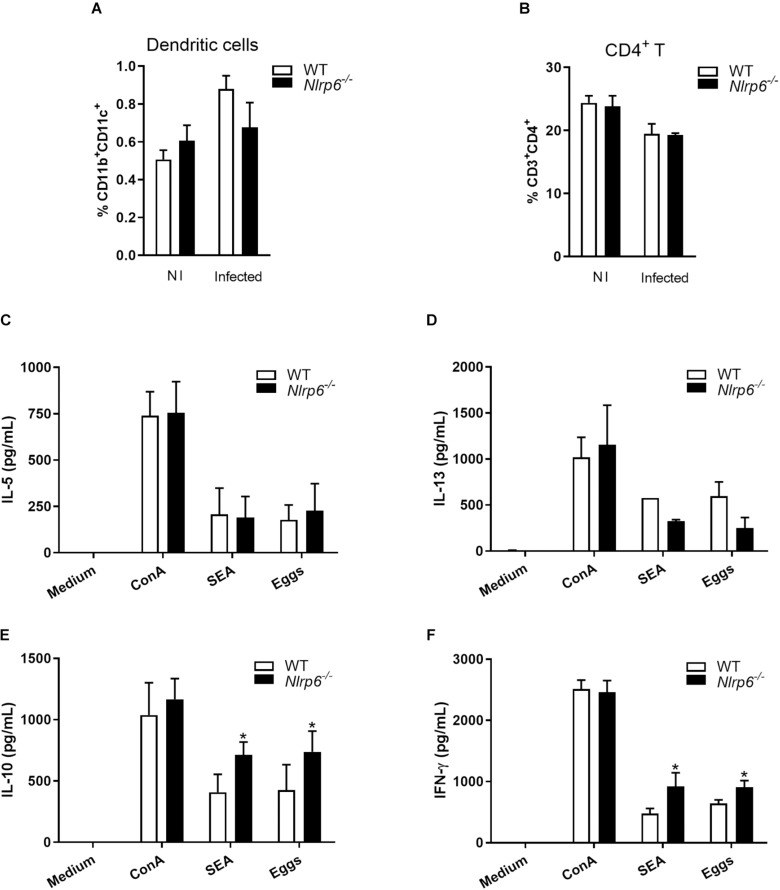
Cytokine profile induced by SEA/Eggs in *Nlrp6*^–/–^ mice. Six-weeks post infection the percentage of **(A)** dendritic cells (CD11b^+^CD11c^+^) and **(B)** CD4^+^ T lymphocytes (CD3^+^CD4^+^) in spleens from non-infected (NI) and infected animals were analyzed by flow cytometry. Spleen cells of infected mice were restimulated with ConA (5 μg/mL), SEA (20 μg/mL) or Eggs (50/well). Cytokine levels were measured by ELISA in cell supernatants from antigen restimulated cells, **(C)** IL-5, **(D)** IL-13, **(E)** IL-10 and **(F)** IFN-γ. An asterisk denotes statistically significant differences between NLRP6 versus WT animals (*p* < 0.05).

After determining the impact of NLRP6 on inflammasome activation and granuloma formation in response to egg antigens, we decided to investigate the frequency of dendritic cells and CD4^+^ T lymphocytes in spleen cells derived from *S. mansoni* infected mice. Cells were obtained following the gate strategy described in Supplementary Figure S3. We observed that 6-week-infected *Nlrp6*^–/–^ and WT mice presented no significant difference regarding CD11b^+^CD11c^+^ (dendritic cells) and CD3^+^CD4^+^ (CD4^+^ lymphocytes) cell populations (Figures 3A,B). Interestingly, when spleen cells were stimulated with eggs or SEA, the cytokine production was altered. Both antigens induced increased levels of IL-10 and IFN-γ in splenocyte culture supernatants from *Nlrp6*^–/–^ compared to WT mice (Figures 3E,F). Additionally, NLRP6 appears to have no effect on IL-5 and IL-13 production (Figures 3C,D). Curiously, when *Nlrp6*^–/–^ and WT spleen cells from *S. mansoni* infected mice were stimulated with SWAP, no significant difference on IFN-γ, IL-10, IL-5, and IL-13 levels was observed (Supplementary Figure S4). These data emphasize the relevance of NLRP6 during egg antigen response regulating IL-10 and IFN-γ production. Furthermore, enhanced IL-10 production in *Nlrp6*^–/–^ may be related to reduced granuloma formation and fibrosis.

### NLRP6 Mediates Innate Immune Cells Recruitment in *Schistosoma*-Infected Liver

**FIGURE 4:**
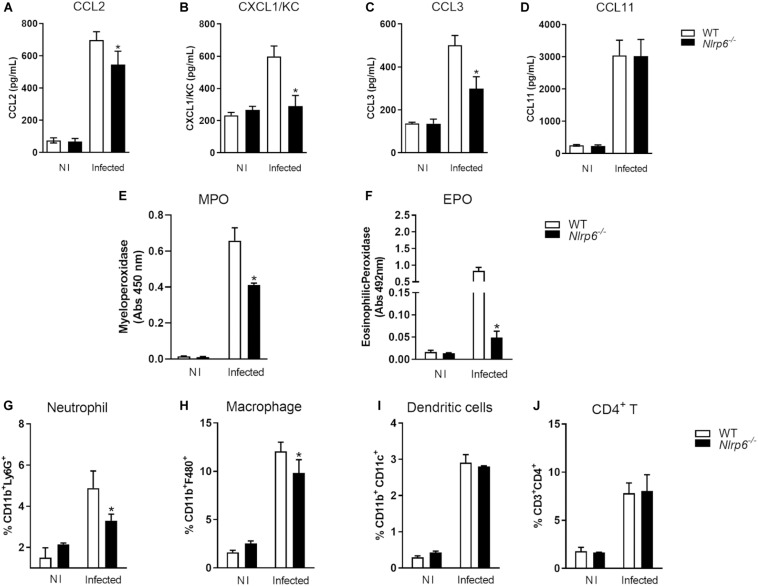
NLRP6 regulates chemokine production and immune cell recruitment into the liver. After 6 weeks of infection, the liver was used to measure chemokine levels, MPO and EPO activities and the frequency of immune cells. Levels of **(A)** CCL2, **(B)** CXCL1 **(C)** CCL3 and **(D)** CCL11 were measured by ELISA. **(E,F)** MPO and EPO activities were also detected. The percentage of **(G)** CD11b^+^Ly6G^+^, **(H)** CD11b^+^F4/80, **(I)** CD11b^+^CD11c^+^ and **(J)** CD3^+^CD4^+^ T lymphocyte populations were measured by flow cytometry. An asterisk denotes statistically significant differences between NLRP6 versus WT animals (*p* < 0.05). NI stands for Non-infected mice.

Since lack of NLRP6 has been shown to modulate hepatic granuloma formation, we decided to investigate how this sensor influences liver pathology. Initially, we evaluated the level of chemokines (CCL2, CCL3, CCL11, and CXCL1) in livers of *Schistosoma*-infected mice. These chemokines have already been described as related to granuloma formation. In *Schistosoma*-infected *Nlrp6*^–/–^ mice only the production of CCL11 was not reduced in comparison to WT mice (Figure 4D). CCL2, CXCL1, and CCL3 were diminished in *Nlrp6*^–/–^ mice compared to WT animals (Figures 4A–C). Additionally, we observed that the enzymes MPO and EPO were also reduced in *Nlrp6*^–/–^ mice when compared to WT (Figures 4E,F). Our next step was to evaluate which non-parenchymal cell populations could be altered in *Schistosoma*-infected *Nlrp6*^–/–^ mice. Cells were obtained following the gate strategy described in Supplementary Figure S5. Neutrophils and macrophages were the major cell populations reduced in *Nlrp6*^–/–^ mice when compared to WT (Figures 4G,H). The frequency of dendritic cells and CD4^+^ T lymphocytes remained unaltered in either mouse groups (Figures 4I,J). We also found that the lack of NLRP6 does not affect macrophage polarization, even though there is a strong tendency of reduction in anti-inflammatory macrophages (CD206^+^CD163^+^) in *Nlrp6*^–/–^ compared to WT mice (Supplementary Figure S6). Therefore, the NLRP6 sensor possibly induces the formation of hepatic granuloma by favoring chemokine production and the recruitment of immune cells to the liver.

### Inflammasome Pathway Is Broadly Relevant to Granuloma Formation

**FIGURE 5:**
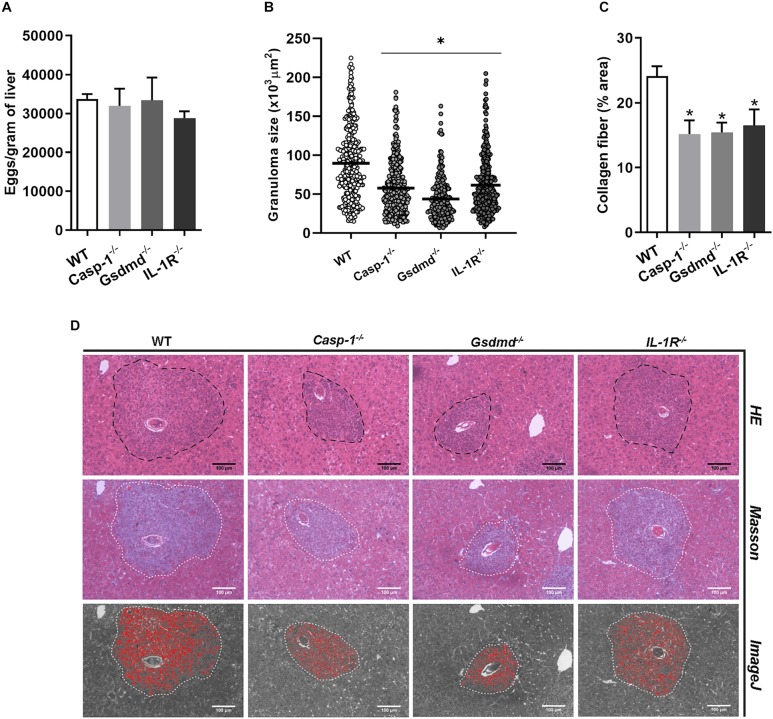
Inflammasome activation influences granuloma formation and collagen deposition. Pathological parameters were analyzed for other inflammasome components such as Casp-1, GSDMD and IL-1R in mouse livers. **(A)** Number of eggs per gram of liver, **(B)** Granuloma size and **(C)** collagen deposition were measured in WT and Casp-1, GSDMD and IL-1R deficient animals. **(D)** Representative images of granulomas detected in hematoxylin and eosin, masson blue and the output from ImageJ software, respectively. An asterisk denotes statistically significant differences between deficient mice versus WT animals (*p* < 0.05). The bar represents 100 μm.

The NLR family receptors perform their functions depending on tissue and cell type, as already demonstrated for NLRP3 and NLRP6 (29, 30). For this reason, we decided to evaluate whether other inflammasome pathway-related molecules, such as Casp-1, GSDMD, and IL-1R, played a role in the formation of hepatic granuloma. Similarly to what we observed here for *Nlrp6*^–/–^ mice, a significant reduction in periovular inflammatory response in *Casp-1^–/–^*, *Gsdmd*^–/–^, and *IL-1R^–/–^* mice was observed. In addition, a reduction in collagen deposition was observed in the granulomas of *Casp-1^–/–^*, *Gsdmd*^–/–^, and *IL-1R^–/–^* mice when compared to WT (Figures 5B–D). This was accompanied by no alteration in the number of eggs in the tissue (Figure 5A), and worm burden recovery (Supplementary Figure S2) when compared to WT mice.

## Discussion

The role of the inflammasome pathway in the pathogenesis of chronic liver diseases has been investigated in the last few years (31, 32). Hepatic injuries from different sources are capable of leading to inflammasome activation, as described for drug-induced damage (33), ischemia-reperfusion (34), alcoholic and non-alcoholic fatty liver disease (15, 16), and viral hepatitis (14). Inflammasome triggers or amplify liver diseases by releasing pro-inflammatory cytokines such as IL-1β, IL-1α, IL-18, and also through other inflammatory mediators such as High Mobility Group Box 1 (HMGB1) (35). Release of such cytokines and DAMPs occur, in part, due to Gasdermin-D cleavage and subsequent pyroptosis of the cell (36). The fibrotic process resulting from chronic liver diseases has also involved the inflammasome pathway. IL-1β and danger signals induce Hepatic Stellate Cells (HSC) to transdifferentiate and perform extracellular matrix remodeling function. In addition, HSCs can internalize pre-formed inflammasome complexes released by other pyroptosis dying cells (37–39).

The participation of the NLRP3 receptor in chronic liver diseases such as non-alcoholic fatty liver disease (NAFLD) is well described (16, 17). On the other hand, the role of the NLRP6 sensor in these liver pathologies is still elusive. Recently, Xiao and colleagues (2018) reported that in the NAFLD model induced by methionine-choline deficient (MCD) diet, NLRP3 and NLRP6 expression is highly detected in the liver. After *Lycium barbarum* polysaccharides (LBP) treatment, NAFLD condition improves and the expression of both NLR receptors decreases (20). Besides, a previous study with NAFLD obese patients demonstrates that when hepatic portal fibrosis is present, the expression of *NLRP6* mRNA in adipose tissues is higher compared to cases when hepatic portal fibrosis is not observed (21). These findings suggest that NLRP6 might play important role in chronic liver disease and fibrosis. All those findings intrigued us to evaluate the role of this sensor in *S. mansoni* infection.

*Schistosoma mansoni* and *S. japonicum* infections are sources of injury and are able to induce chronic liver disease. The long survival period of *Schistosoma* worms within the human host implies recurrent inflammation and wound-healing cycles in the liver, triggered by egg antigens which can result in fibrosis, portal hypertension and hepatosplenomegaly (4, 8). Among non-parenchymal liver cells, DCs stand out as key cells during this pathological process. Broadly responsive to egg antigens, DCs are essential for promoting systemic shift in the immune response profile (from Th1 to Th2), which is crucial for host survival upon infection (28, 40). In addition, the first report of inflammasome pathway activation by *S. mansoni* antigens involved dendritic cells responding to SEA. For these reasons, we initially decided to evaluate the role of NLRP6 in DCs. Our findings suggest that NLRP6 is important for the formation of the inflammasome complex, since it influences IL-1β secretion and caspase-1 activation in response to SEA. Previous studies demonstrate that NLRP6 structurally has the ability to form the inflammasome complex and does so in response to gram-positive bacteria cell wall components, activating both caspase-1 and caspase-11 in the same complex (41, 42).

The activation of intracellular receptors was not expected to occur in response to multicellular parasites such as *S. mansoni*. Surprisingly, Ritter and colleagues (2010) demonstrated that SEA triggers NLRP3 inflammasome pathway in DCs (9). Following this seminal study, NLRP3 inflammasome role has been investigated, especially in *S. japonicum* infection. In this context, NLRP3 has been shown to be pivotal for inducing hepatic granuloma formation and collagen deposition in the granulomatous structure (18, 43). In this study, we confirmed that NLRP3 is pivotal for hepatic granuloma formation, but we did not find any alteration in collagen deposition in *Nlrp3*^–/–^ mice when compared to WT (Supplementary Figure S7). This observation may be related to the early time point infection of our model (6 weeks), which also could explain the distribution of collagen throughout the granuloma structure and not only peripherally, as typically observed in later granulomas. Surprisingly, we have demonstrated for the first time that NLRP6 also plays an important role in *S. mansoni*-induced pathology. We found that this sensor influences the formation of hepatic granuloma, altering local chemokine (CCL2, CCL3, and CXCL1) and cytokine (IL-5, IL-10, and IL-13) production, macrophage and neutrophil recruitment into the liver, and also is important for promoting collagen deposition. In *Schistosoma* egg-induced pathology, chemokine production is essential to modulate granuloma formation (44, 45). CCL3-deficient mice, for instance, showed size reduced granuloma, lower fibrosis and lower EPO activity in the liver (46). Although classically responsible for neutrophil recruitment, CXCL1 also impacts on the recruitment of HSCs, which are responsible for collagen deposition in the granuloma structure (47). Following injury, hepatic resident cells produce CCL2, important for monocyte and macrophage recruitment (48).

NLRP6 is known as an atypical sensor with wide functional capability, performing activities integrated and/or independent on the inflammasome complex (30). Our findings demonstrate that Casp-1 activation and IL-1β production, in response to SEA, can be regulated by NLRP6. Therefore, our findings suggest that the role of this receptor in hepatic granuloma formation is due to the inflammasome activation, since *Nlrp3*^–/–^ mice have demonstrated a similar phenotype. It is clear that the inflammasome pathway is important for *Schistosoma*-induced liver pathology, once we have observed granuloma reduction and lower collagen deposition in *Casp-1^–/–^*, *Gsdmd*^–/–^, and *IL-1R^–/–^* mice when compared to WT. Previous liver disease studies support our observations for *Casp-1^–/–^* and *IL-1R^–/–^* mice. In a high fat diet-induced NASH model, deficient Casp-1 animals showed improvement in hepatic steatosis, inflammation and fibrogenesis (49). Similarly, IL-1R signaling has been shown to be critical for the progression of steatohepatitis and hepatic fibrosis in hypercholesterolemic mice (50). The role of Gasdermin- D and pyroptosis has also been described in chronic liver diseases (51, 52). During human NAFLD/NASH, GSDMD and its N-terminal peptide (GSDMD-N) are upregulated, besides MCD-fed *Gsdmd*^–/–^ mice showed decreased severity of steatosis and inflammation comparing to WT (52). During *Schistosoma* infection, Liu and colleagues (2019) demonstrated that *S. japonicum* induces expression of the GSDMD-N in the liver, and that this expression is modulated by NLRP3 sensor (53). In addition, it has been reported that SEA from *S. japonicum* eggs induces pyroptosis in HSCs (54). However, no *in vivo* mouse study has been reported correlating GSDMD deficiency and granuloma formation and fibrosis induced by *S. mansoni* infection as demonstrated here.

In summary, the data presented here demonstrate that lack of NLRP6 modulates activation of the inflammasome pathway in response to *S. mansoni* egg antigens. In addition, NLRP6 and the inflammasome components are important in liver pathology induced by *S. mansoni* infection. Taken together, these data reinforce the relevance of understanding the inflammasome signaling pathway, given its potential to influence the severe pathological conditions induced by this disease.

## Data Availability Statement

All datasets generated for this study are included in the article/Supplementary Material.

## Ethics Statement

The animal study was reviewed and approved by the Committee on Ethics of Animal Experiments of the Federal University of Minas Gerais (Permit Number: #367/2017) and carried out in accordance with Brazilian laws #6638 and #9605 in Animal Experiments.

## Author Contributions

RS and SO designed the project and experiments, and wrote the manuscript. RS, CS, FVM, FSM, EG, and SM carried out most of the experiments. RS carried out statistical analysis and prepared the figures. SO submitted this manuscript. All authors reviewed the manuscript.

## Conflict of Interest

The authors declare that the research was conducted in the absence of any commercial or financial relationships that could be construed as a potential conflict of interest.

## Footnotes

1 http://rsbweb.nih.gov/ij/index.html
