# Supplementary material for: NLRP6 Plays an Important Role in Early Hepatic Immunopathology Caused by Schistosoma mansoni Infection
Source: Front Immunol. 2020 May 5;11:795. doi: 10.3389/fimmu.2020.00795 (PMC7214731; doi:10.3389/fimmu.2020.00795)
Supplement: Supplementary file 1 [file Image_1.pdf]

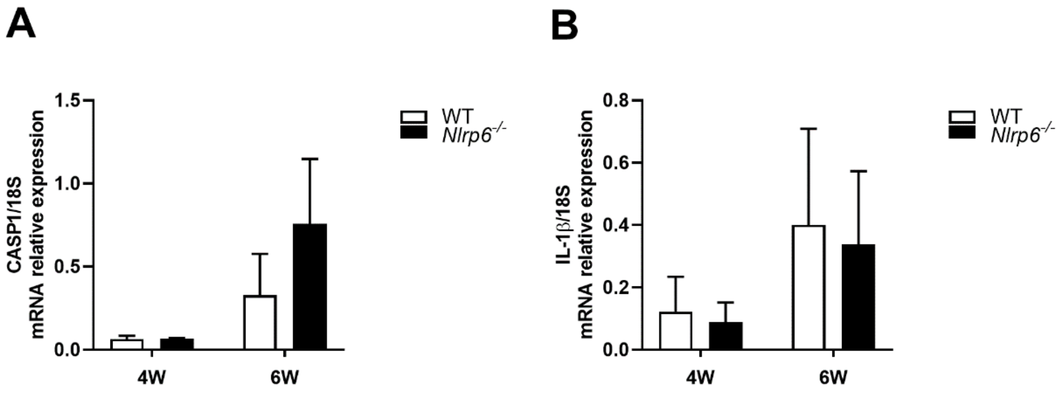

Supplementary Figure 1. Casp-1 and IL-1β mRNA levels during *S. mansoni* infection. After four (4W) and six (6W) weeks of infection, livers from WT and *Nlrp6*$^{-/-}$ mice were collected. RNA was extracted and qPCR assay conducted for (A) *Caspase-1* and (B) *IL-1β*.
