# Supplementary material for: NLRP6 Plays an Important Role in Early Hepatic Immunopathology Caused by Schistosoma mansoni Infection
Source: Front Immunol. 2020 May 5;11:795. doi: 10.3389/fimmu.2020.00795 (PMC7214731; doi:10.3389/fimmu.2020.00795)
Supplement: Supplementary file 2 [file Image_2.pdf]

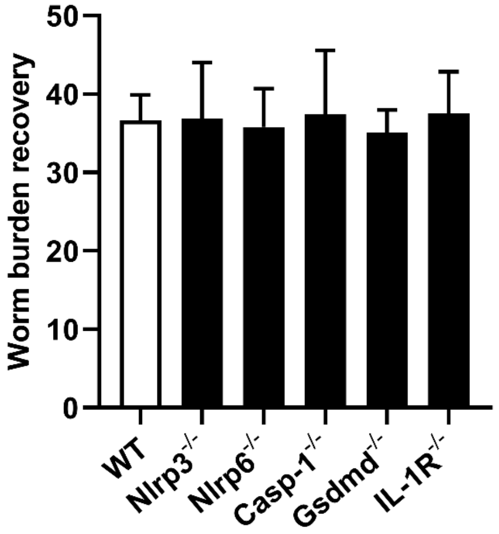

Supplementary Figure 2. Worm burden recovery from NLRP3, NLRP6, caspase-1, Gasdermin-D and IL-1R knockout mice. After six weeks of infection (100 cercariae), mice were euthanized and perfused from the portal veins. The recovered worms were counted and the mean difference between groups of mice was evaluated. Graphs represent data from two independent trials.
