# Supplementary material for: NLRP6 Plays an Important Role in Early Hepatic Immunopathology Caused by Schistosoma mansoni Infection
Source: Front Immunol. 2020 May 5;11:795. doi: 10.3389/fimmu.2020.00795 (PMC7214731; doi:10.3389/fimmu.2020.00795)
Supplement: Supplementary file 3 [file Image_3.pdf]

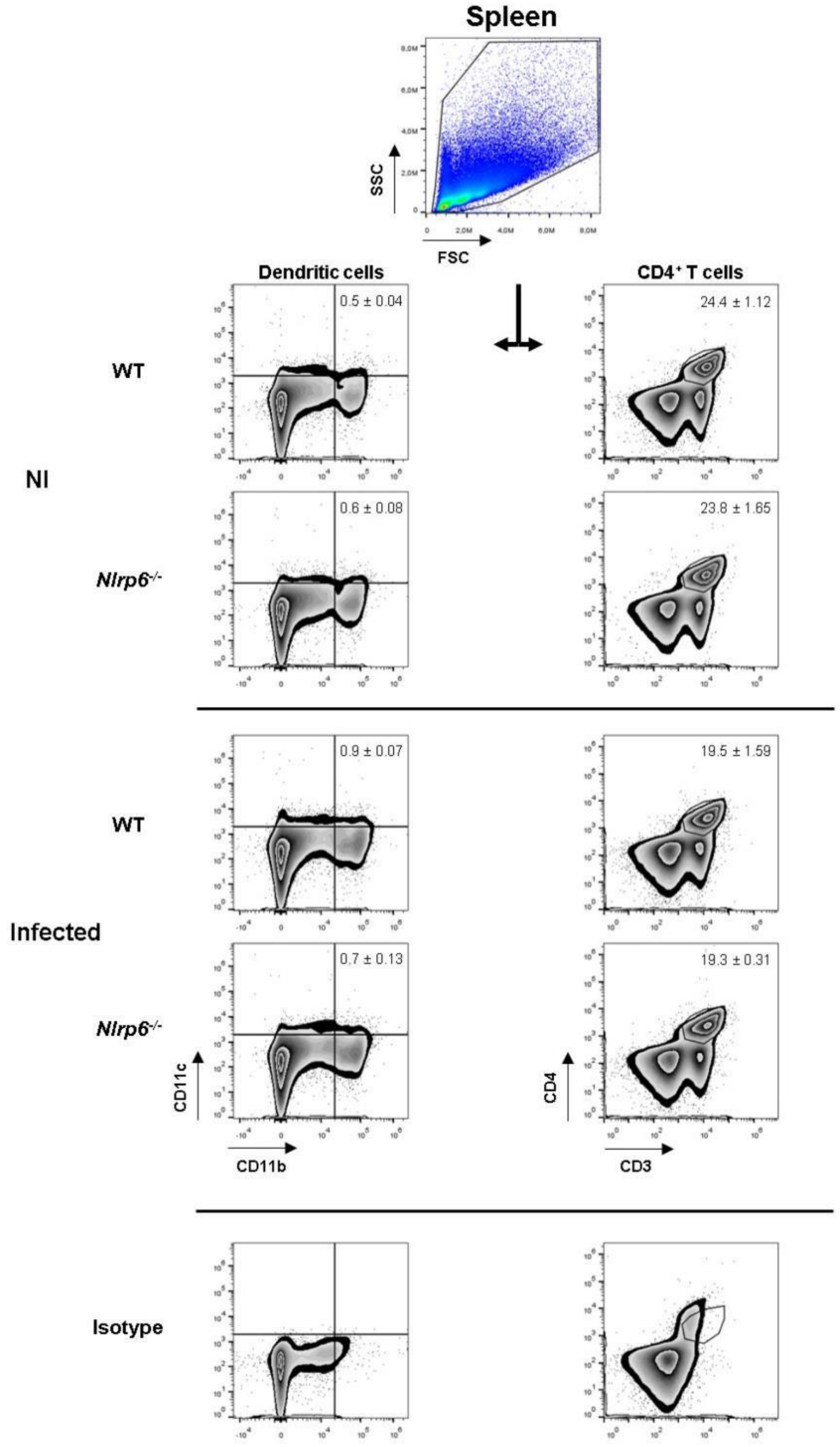

Supplementary Figure 3. Flow cytometry plot analysis of WT and *Nlrp6*$^{-/-}$ mice spleen. After six weeks of infection, non-infected (NI) and infected splenocytes were stained *ex vivo* for surface markers. The results presented in Figure 3 (A and B) were analyzed as follows: A gate in SSC-A and FSC-A was performed followed by a selection of CD11b^{+}CD11c^{+} and CD3^{+}CD4^{+} double-positive cells. As negative control, cells were stained with isotype controls for each mix of antibodies.
