# Supplementary material for: NLRP6 Plays an Important Role in Early Hepatic Immunopathology Caused by Schistosoma mansoni Infection
Source: Front Immunol. 2020 May 5;11:795. doi: 10.3389/fimmu.2020.00795 (PMC7214731; doi:10.3389/fimmu.2020.00795)
Supplement: Supplementary file 4 [file Image_4.pdf]

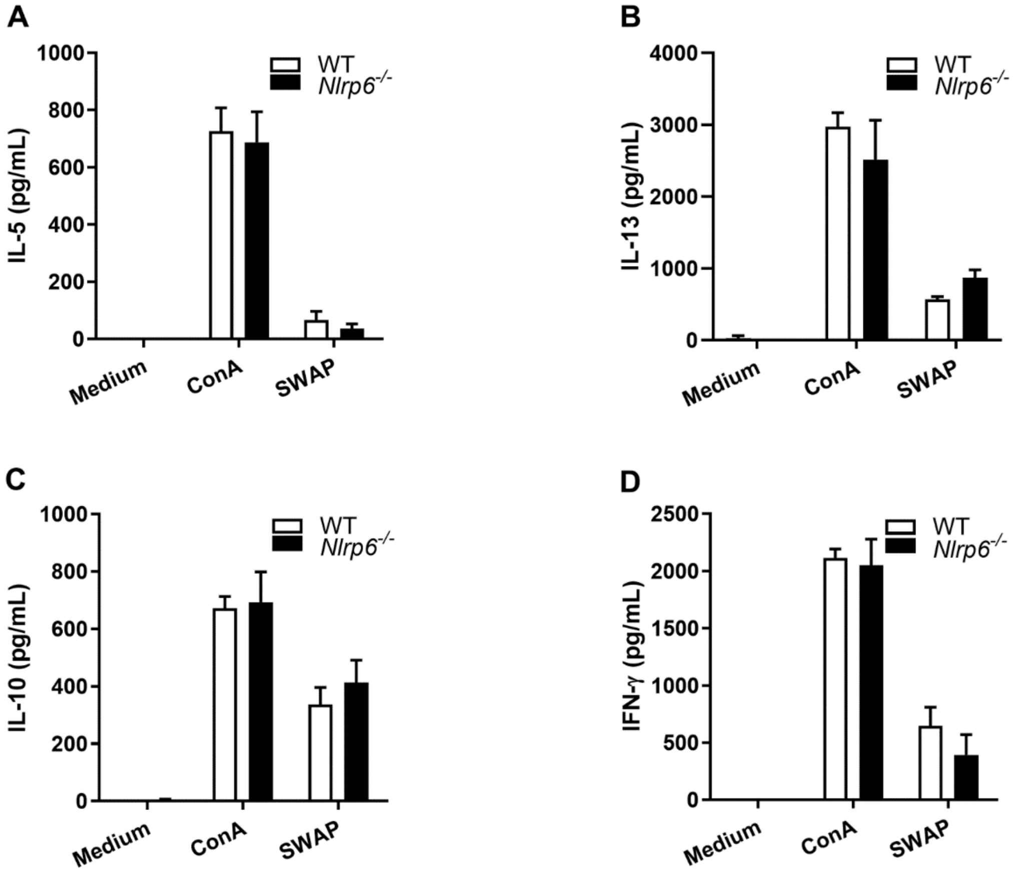

Supplementary Figure 4. SWAP does not alter spleen cytokine production in *Nlrp6*$^{-/-}$ mice. Six-weeks post infection spleens were obtained and splenocytes were cultured. Cells were restimulated with ConA (5 µg/mL) or SWAP (200 µg/mL). Cytokines levels were measured by ELISA in supernatant, 24h (A) and 72h (B, C, D) after stimuli.
