# Supplementary material for: NLRP6 Plays an Important Role in Early Hepatic Immunopathology Caused by Schistosoma mansoni Infection
Source: Front Immunol. 2020 May 5;11:795. doi: 10.3389/fimmu.2020.00795 (PMC7214731; doi:10.3389/fimmu.2020.00795)
Supplement: Supplementary file 5 [file Image_5.pdf]

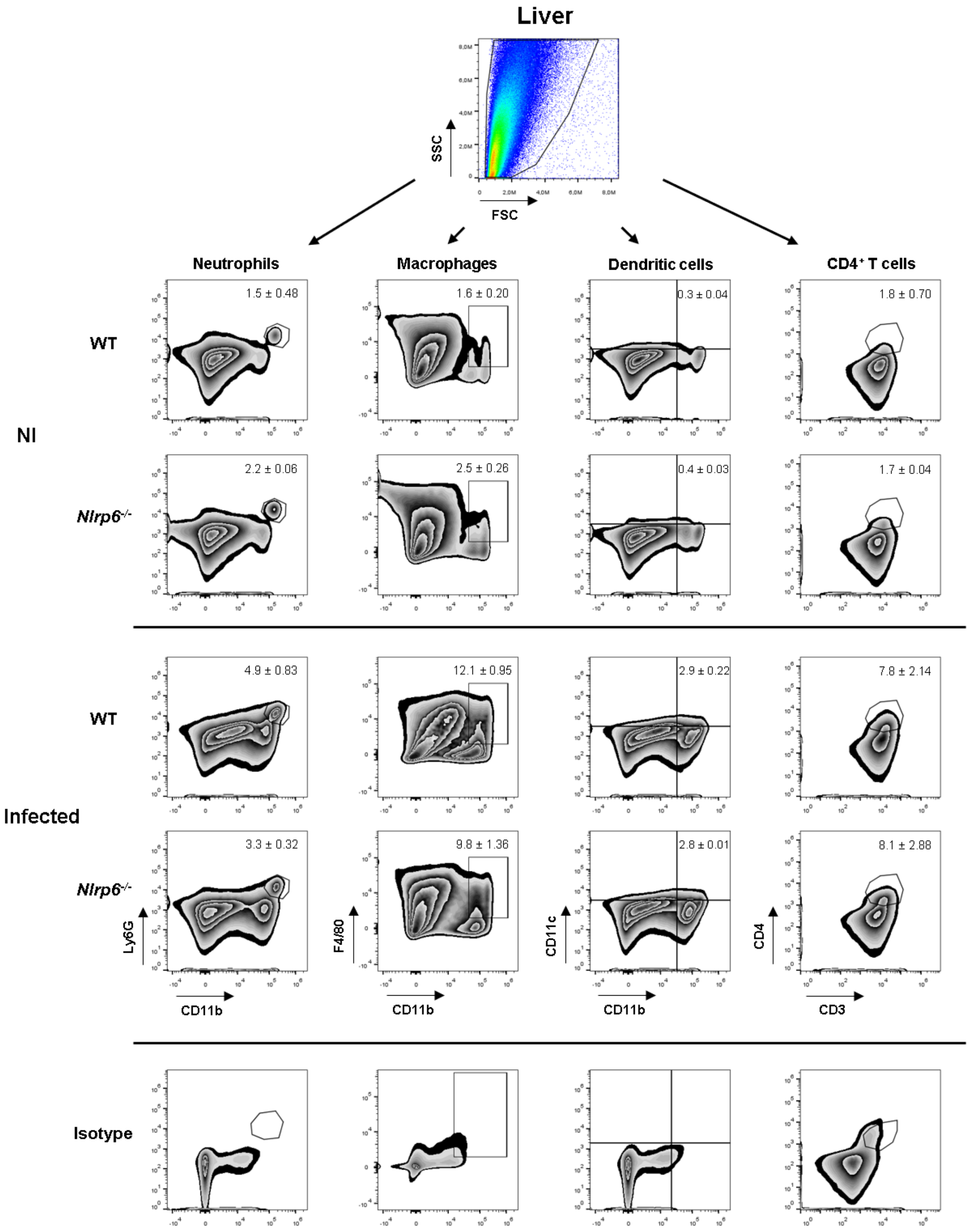

Supplementary Figure 5. Flow cytometry plots of WT and *Nlrp6*$^{-/-}$ mice liver. Non-infected (NI) and infected non-parenchymal liver cells were stained *ex vivo* for surface markers, after six weeks of infection. The results presented in Figure 4 (G-J) were analyzed as follows: A gate in SSC-A and FSC-A was performed, followed by a selection of CD11b^{+}Ly6G^{+}, CD11b^{+}F4/80^{+}, CD11b^{+}CD11c^{+} and CD3^{+}CD4^{+} double-positive cells. As negative control, cells were stained with isotype controls for each mix of antibodies.
