# Supplementary material for: NLRP6 Plays an Important Role in Early Hepatic Immunopathology Caused by Schistosoma mansoni Infection
Source: Front Immunol. 2020 May 5;11:795. doi: 10.3389/fimmu.2020.00795 (PMC7214731; doi:10.3389/fimmu.2020.00795)
Supplement: Supplementary file 6 [file Image_6.pdf]

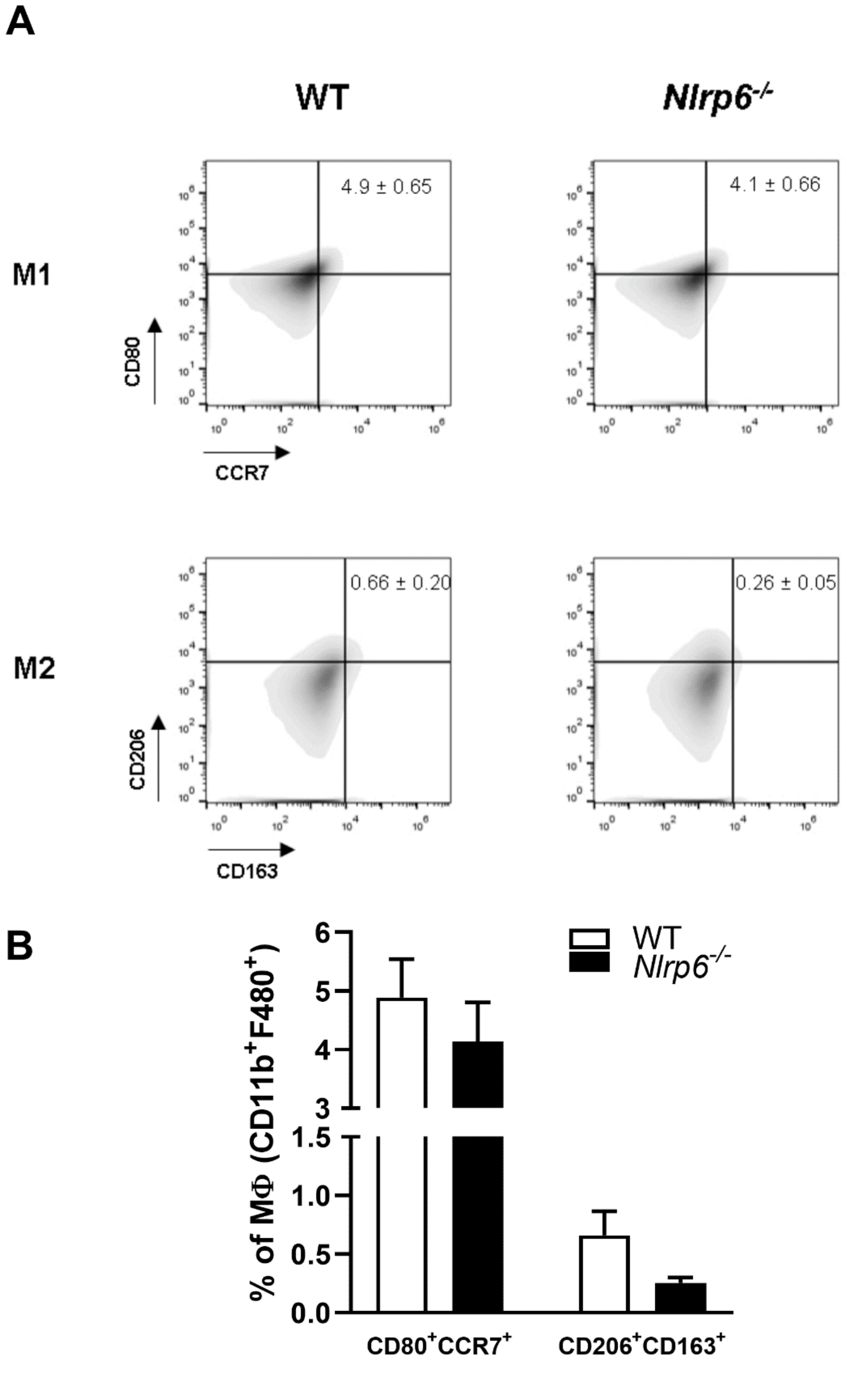

Supplementary Figure 6. Analysis of macrophage polarization in the liver. Expression of M1 (CD80$^+$CCR7$^+$) and M2 (CD206$^+$CD163$^+$) markers were determined by flow cytometry in non-parenchymal liver cells, collected after six weeks of infection. CD11b$^+$F4/80$^+$ cells were pre-gated as previously described in gate strategy for liver non-parenchymal cells. CD80$^+$CCR7$^+$ and CD206$^+$CD163$^+$double positive cells were selected, as shown on representative dot plots (A), together with cell frequency (B).
