# Supplementary material for: NLRP6 Plays an Important Role in Early Hepatic Immunopathology Caused by Schistosoma mansoni Infection
Source: Front Immunol. 2020 May 5;11:795. doi: 10.3389/fimmu.2020.00795 (PMC7214731; doi:10.3389/fimmu.2020.00795)
Supplement: Supplementary file 7 [file Image_7.pdf]

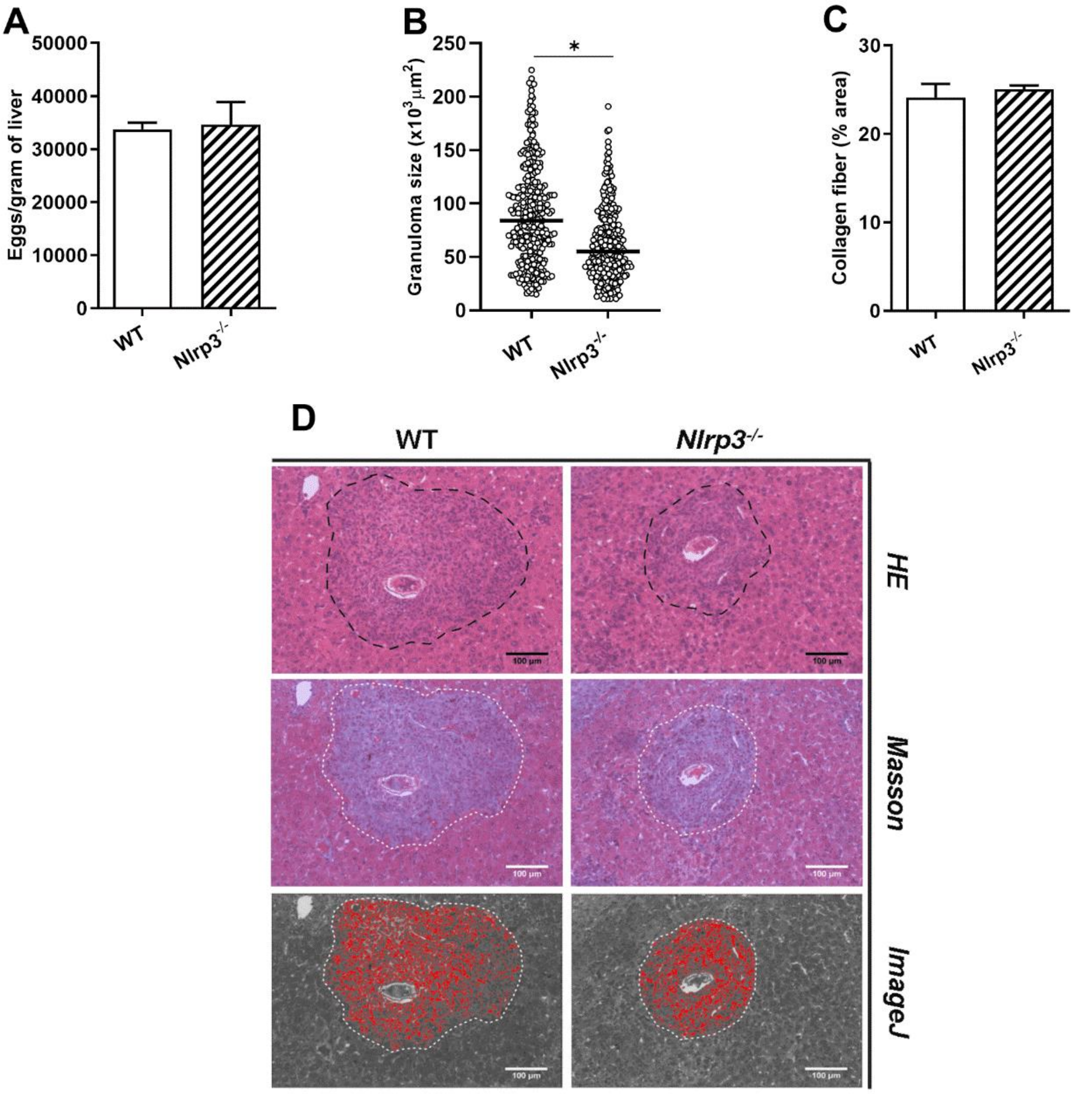

Supplementary Figure 7. NLRP3 sensor influences *Schistosoma*-induced immunopathology. WT and *Nlrp3*$^{-/-}$ mice pathological parameters were evaluated in different liver portions, after six weeks of infection. (A) Number of eggs per gram of liver, (B) Granuloma size (μm^2) and (C) Collagen deposition. (D) Representative images of granulomas detected in hematoxylin-eosin, masson blue and the output from ImageJ software, respectively. An asterisk denotes statistically significant differences between NLRP3 versus WT animals ($p < 0.05$). The bar represents 100 µm.
